# Editorial: The role of AI in GU oncology

**DOI:** 10.3389/fonc.2025.1588967

**Published:** 2025-03-24

**Authors:** Sungun Bang, Kazumi Taguchi, Martin King, Kyo Chul Koo

**Affiliations:** ^1^ Department of Urology, Yonsei University College of Medicine, Seoul, Republic of Korea; ^2^ Department of Urology, Graduate School of Medical Sciences, Nagoya City University, Nagoya, Japan; ^3^ Department of Urology, Brigham and Women’s Hospital, Harvard Medical School, Boston, MA, United States

**Keywords:** artificial intelligence, pattern recognition, data analysis, imaging interpretation, pathologic interpretation

While significant progress has been made in detection, diagnosis, and treatment of genitourinary (GU) cancers, the impact of artificial intelligence (AI) in this field is still being explored. AI has significantly enhanced efficiency in healthcare, particularly in histopathology assessment, tumor detection, diagnostic imaging, risk stratification, and personalized treatment. Early skepticism about AI in medicine has diminished with advancements in computational power, leading to clinically useful platforms over the past two decades. This Research Topic examined the role of AI in GU oncology, focusing on complex medical data analysis, imaging and pathological data interpretation, and data monitoring for pattern recognition.

Bang et al. reviewed the application of machine learning (ML) in predicting survival outcomes in prostate cancer (PCa). The study highlighted the role of ML in forecasting biochemical recurrence-free, progression to castration-resistance-free, metastasis-free, and overall survivals using pathological, radiological, and electronic medical record data. These advancements demonstrate that ML-driven prediction models can match or surpass traditional statistical methods. Abbas et al. explored ML’s ability to improve non-muscle-invasive bladder cancer (NMIBC) recurrence prediction by integrating radiomics, clinical, and genomic data across various ML models, including neural networks, deep learning (DL), and random forest. Their findings suggest that multi-modal ML models outperform conventional methods, though challenges such as limited generalizability, small datasets, and model opacity hinder clinical adoption. The study provides strategies to optimize ML models, improve data integration, and enhance real-world applicability in NMIBC care. Du et al. assessed machine learning (ML) models for predicting prostate-specific antigen (PSA) persistence after radical prostatectomy. Among seven ML algorithms tested on 470 patients, random forest performed best, with an AUC of 0.861 in the training set and 0.801 in the test set. The most important predictors were capsular invasion, positive surgical margin, preoperative PSA, and biopsy Gleason score. This study underscores the potential of random forest in improving PSA persistence prediction and aiding timely treatment planning. Kim et al. examined computational methods for spatially mapped omics data analysis using digitized histopathology slides. Their review covered image processing techniques, ML integration with spatially resolved omics data, and the challenges of incorporating ML into clinical decision-making. Ma et al. analyzed the role of AI in bladder cancer (BC) diagnosis, treatment, and prognosis. The study highlighted ML, DL, and artificial neural networks as transformative tools that enhance early detection, diagnostic accuracy, and personalized treatment while predicting disease progression. At the same time, they bring up challenges, including data acquisition, standardization, and ethical concerns. Halawani et al. evaluated the accuracy and readability of AI-generated patient education materials (PEMs) on kidney cancer using ChatGPT-4.0, Gemini AI, and Perplexity AI, comparing them to PEMs from the American Urological Association and the European Association of Urology. While AI models seem to generate and simplify PEMs, the authors conclude that their inconsistencies and potential inaccuracies make them best suited as supplementary tools rather than primary educational resources. Zhu et al. reviewed ML and DL applications in urological tumors, assessing the role of AI in clinical decision-making and urological surgery. The study suggests that AI is poised to play an increasingly significant role in diagnosis, treatment planning, and rehabilitation monitoring. Despite current limitations, AI holds promise for improving personalized care, patient survival rates, and quality of life in GU oncology. Drożdż et al. applied ML to classify hematuria patients and identify key biomarkers. Among the models tested, the Comprehensive Abstraction and Classification Tool for Uncovering Structures (CACTUS) algorithm outperformed decision trees and random forests, achieving 80% accuracy for males. The most relevant biomarkers were microalbumin, total PSA, and gender, with additional markers varying by sex. This study highlights CACTUS as a reliable tool for unbalanced datasets and suggests these biomarkers could improve personalized hematuria diagnosis.

Overall, challenges seem to remain in the utilization of AI in improving patient care. For a more precise ML-based algorithm, key strategies should be employed, including 1) enhancing the quality of data preparation, 2) selecting an optimal algorithm aligned with the study objective, 3) optimizing hyperparameters, 4) refining model architecture, 5) addressing overfitting and underfitting issues, and 6) improving computational power. When integrating ML models into real-world clinical practice, it is critical to address biases and inequalities, particularly those arising from algorithms trained on heterogeneous and ethnically diverse PCa populations. External validation across diverse populations and medical centers can improve the reliability and generalizability of these models, facilitating their inclusion in clinical guidelines and routine practice.

We sincerely thank all contributing authors, reviewers, and researchers for their dedication to advancing AI in oncology. Special thanks to Professors Kazumi Taguchi and Martin King for their contributions as co-editors. Their expertise has been instrumental in shaping this Research Topic, and we look forward to seeing these findings inspire future advancements in AI-driven GU oncology.

